# Kaempferol, a Major Flavonoid in Ginkgo Folium, Potentiates Angiogenic Functions in Cultured Endothelial Cells by Binding to Vascular Endothelial Growth Factor

**DOI:** 10.3389/fphar.2020.00526

**Published:** 2020-04-28

**Authors:** Wei-Hui Hu, Huai-You Wang, Yi-Teng Xia, Diana Kun Dai, Qing-Ping Xiong, Tina Ting-Xia Dong, Ran Duan, Gallant Kar-Lun Chan, Qi-Wei Qin, Karl Wah-Keung Tsim

**Affiliations:** ^1^Shenzhen Key Laboratory of Edible and Medicinal Bioresources, The Hong Kong University of Science and Technology, Nanshan, Shenzhen, China; ^2^Division of Life Science and State Key Laboratory of Molecular Neuroscience, The Hong Kong University of Science and Technology, Hong Kong, Hong Kong; ^3^Joint Laboratory of Guangdong Province and Hong Kong Region on Marine Bioresource Conservation and Exploitation, College of Marine Sciences, South China Agricultural University, Guangzhou, China; ^4^Jiangsu Key Laboratory of Regional Resource Exploitation and Medicinal Research, Huaiyin Institute of Technology, Jiangsu, China

**Keywords:** kaempferol, *Ginkgo biloba*, VEGF, angiogenesis, herbal medicine

## Abstract

Kaempferol is a major flavonoid in Ginkgo Folium and other edible plants, which is being proposed here to have roles in angiogenesis. Angiogenesis is important in both physiological and pathological development. Here, kaempferol was shown to bind with vascular endothelial growth factor (VEGF), probably in the heparin binding domain of VEGF: this binding potentiated the angiogenic functions of VEGF in various culture models. Kaempferol potentiated the VEGF-induced cell motility in human umbilical vein endothelial cells (HUVECs), as well as the sub-intestinal vessel sprouting in zebrafish embryos and formation of microvascular in rat aortic ring. In cultured HUVECs, application of kaempferol strongly potentiated the VEGF-induced phosphorylations of VEGFR2, endothelial nitric oxide synthase (eNOS) and extracellular signal-regulated kinase (Erk) in time-dependent and concentration-dependent manners, and in parallel the VEGF-mediated expressions of matrix metalloproteinases (MMPs), MMP-2 and MMP-9, were significantly enhanced. In addition, the potentiation effect of kaempferol was revealed in VEGF-induced migration of skin cell and monocyte. Taken together, our results suggested the pharmacological roles of kaempferol in potentiating VEGF-mediated functions should be considered.

## Introduction

It has been proved that angiogenesis is defined by the formation of new vessels from pre-existing blood vessels in the blood vascular system (Folkman and D'Amore, 1996), which is contributing a number of physiologic and pathologic processes, including wound healing, embryo development, malignancy, and chronic inflammation (Folkman, 1995; Carmeliet and Jain, 2000). The process of vessel formation is highly dynamic and complicated, which controls a large quantity of pro-angiogenic molecules and/or anti-angiogenic molecules (Rafii et al., 2002). Several steps are required during angiogenesis, including endothelial cell proliferation and migration, sequential degradation of basement membrane, and new vessel stabilization: the modulation of these steps potentiates, or suppresses, new blood vessel formation. A large number of growth factors and related receptors focusing on angiogenesis have been identified. Among these known angiogenic factors, vascular endothelial growth factor (VEGF) is a major regulator of angiogenesis acting as a major pro-angiogenic factor (Ferrara, 1996). VEGF has six members, i.e. from VEGF-A to VEGF-E and placenta growth factor. By binding to its receptors, i.e. from VEGF receptor 1 (VEGFR1) to VEGFR3, the VEGF members are responsible for the regulation of vascular functions. VEGF-A (corresponding to VEGF thereafter) and VEGFR2 are major players in angiogenesis.

Angiogenesis plays vital role in providing tissue regeneration, or sites of repair, with sufficient amounts of nutrient, oxygen and various growth factor: this process is also pre-requisite of removing waste products (Griffith and Naughton, 2002). Speeding up the angiogenic process should be an excellent way to repair tissue damages, e.g. caused by ischemia, and which therefore provides a strong support to development of therapeutic neovascularization (Timar et al., 2001; Emanueli and Madeddu, 2005). In having growth factor as therapeutic agent, the pharmacological activity of growth factor however fails to last very long *in vivo* due to its poor stability, especially those protein-type growth factors. Besides, the injection of high doses of protein-type angiogenic factors might induce side effects. Thus, the search on natural compounds having regulatory roles in angiogenesis could be a possible direction.

Traditional Chinese medicine (TCM) is an excellent source in finding new therapies for different diseases. Ginkgo Folium is a commonly used medicinal herb, which is known to contain a rich source of flavonoidic compounds. Kaempferol, named 3,4′,5,7-tetrahydoxyflavone, is highly enriched in Ginkgo Folium, and indeed this flavonoid is serving as one of the indicative chemicals in assessing quality of Ginkgo Folium, according to Chinese Pharmacopoeia (2015). Kaempferol has been demonstrated to have pharmacological activities, e.g. reducing mortality caused by coronary heart disease and decreasing myocardial infarction incidence (Hertog et al., 1993), inducing antioxidant activities by promoting expression of enzymes related with antioxidant effects (dismutase, heme oxygenase-1 and catalase) (Lin et al., 2003; Hong et al., 2009), inhibiting NF-κB activity for anti-inflammation effects (Wang et al., 2006), inducing osteoblastic differentiation (Guo et al., 2012), and weakening the damage of cigarette smoke in promoting immortalized lung epithelial cell growth (Puppala et al., 2007).

By using HerboChips as a drug screening platform, we have identified polydatin (Hu et al., 2019a) and resveratrol (Hu et al., 2019b) for its binding to VEGF; both of them are deriving from a TCM herb, Polygoni Cuspidati Rhizoma et Radix (Hu et al., 2018). The high affinity binding of polydatin and/or resveratrol to VEGF suppressed the angiogenic effects of VEGF, i.e. decreased the binding of VEGF to its receptors. In the screening of HerboChips, Ginkgo Folium was identified to be one of the positive hits in binding to VEGF. Further screening and fractionation of Ginkgo Folium, kaempferol was identified to bind VEGF; however, this binding, in contrast to polydatin and resveratrol, increased the angiogenic effects of VEGF both *in vitro* and *in vivo*. In this study, we revealed the pharmacological properties of kaempferol in affecting the signaling triggered by VEGF.

## Materials and Methods

### Cell Lines and Chemicals

Human umbilical vein endothelial cells (HUVECs) were bought from Lonza (San Diego, CA): cell passages from three to six were used. Endothelial cells were cultured in EGM-2^®^ BulletKit media according to the instructions (Lonza). RAW 264.7 cells were from American Type Culture Collection (ATCC, Manassas, VA) and human keratinocyte cell line (HaCaT) was obtained from Cell Lines Service (Heidelberg, Germany). RAW 264.7 cells were cultured in Dulbecco's modified Eagle's medium supplemented with 1% streptomycin, 1% penicillin, and 10% heat in-active fetal bovine serum (FBS), while HaCaT cells were cultured in Dulbecco's modified Eagle's medium added with 1% streptomycin, 1% penicillin, and 10% FBS. HUVECs, RAW264.7 cells, and HaCaT cells were maintained in a humidified atmosphere supplying 5% CO_2_ and the temperature was set at 37°C. Human protein (VEGF_165_) was recombinant and was obtained from R&D systems (Minneapolis, MN) and the following antibodies: matrix metalloproteinase (MMP)-2, MMP-9, phospho-p44/42 MAPK [extracellular signal-regulated kinase (Erk) 1/2] (Thr202/Tyr204), p44/42 MAPK (Erk1/2), phospho-endothelial nitric oxide synthase (eNOS) (Ser1177), and eNOS were from Cell Signalling Technology (Danvers, MA); glyceraldehyde 3-phosphate dehydrogenase (GAPDH) antibody and dexamethasone were purchased from Sigma-Aldrich (St. Louis, MO). Kaempferol was supplied by Testing Laboratory for Chinese Medicine of HKUST, and its purity was more than 98% analyzed by HPLC-DAD. For preparation of a stock solution of kaempferol, it was freshly dissolved in dimethyl sulfoxide (DMSO) with the concentration set at 100 mM.

### Immunoprecipitation Assay

Kaempferol solution (100 µl), at a concentration of 0.5 µM, was reacted with protein or biotinylated protein with or without heated at 95°C for 10 min. The reaction lasted for 1 h at 4°C. Then, the reacted solution was independently reacted with 100 µl of PureProteome Streptavidin Magnetic Beads. The binding reaction lasted for 24 h at 4°C. A magnetic stand was used to make the beads gathered at the magnet. Then, without touching the beads, the supernatant was gently gathered, and the VEGF complex and biotin-labeled VEGF complex were separately precipitated with acetonitrile (ACN) after washed with PBS solution for three times. The solution was injected into UPLC instrument equipped with an Agilent, Grace VisionHT C18 column (4.6 × 250 mm, 5 μm) to measure kaempferol's amount. The mobile phase was optimized as below: A, 0.1% formic acid in ACN; B, 0.2% diluted aqueous formic acid, and the optimized gradient elution was as followed: Solvent A was gradually increased from 8% to 18% by 12 min, from 18% to 24% by 24 min, from 24% to 25% by 5 min, from 25% to 40% by 15 min and finally increased from 40% to 75% by 96 min. The determined flow rate was 1 ml/min and the sample volume for injection was 10 μl. The sample temperature was maintained at room temperature. The column temperature was at 30°C. The wavelength of detector was at 360 nm.

The mass spectrometric detection was performed together with a Synapt™ quadrupole time-of-flight (Q-TOF) High-Definition Mass Spectrometer (Waters), equipped with an electrospray ionization source operated under positive ionization mode. The mobile phase consisted of 0.1% formic acid in ACN (A) and 0.2% diluted aqueous formic acid (B). The linear gradient of solvent was same as UPLC analysis, and there were two cycles of weak (90% ACN) and strong (20% ACN) solvent washing of the injecting system between different injections. The mass spectrometric parameters were determined as follows: capillary voltage, 2.5 kV; sample cone, 25 V; extraction cone, 4.0 V; source temperature, 120°C; and desolvation temperature at 350°C. Nitrogen was used in desolvation, and a cone gas was determined at a flow rate of 600 and 50 L/h, respectively. Argon acted as a collision gas. The sample was scanned and analyzed in full-scan mode with m/z set from 80 to 800 in 1-s scan interval. The sample was scanned and analyzed in full-scan mode with m/z set from 80 to 800 in 1-s scan interval in high-resolution mode.

### Molecular Docking

Protein (VEGF) structure was downloaded from Protein Data Bank (PDB), and kaempferol (PubChem: 5280863) was from NCBI-PubChem database. Before analysis, the structure of kaempferol was shifted into MOL2 mode with Chemoffice 2014 software (Cambridge Soft, Cambridge, MA), and the docking analysis was performed based on kaempferol-VEGF protein model. 40 × 40 × 40 Å grid points, as affinity (grid) maps, along with x, y, and z as well as 0.375 Å spacing, were optimized by application of AutoGrid program. The box center was optimized. The value of x was: 0.38 Å, y was −2.98 Å and z was 20.51 Å (Morris et al., 1998). The majority of binding modes were produced for possible bindings, and the energies could be roughly estimated (Grosdidier et al., 2011). The binding interactions were run using vina software; while values representing binding intensity were demonstrated under a PyMOL molecular graphic system.

### Cell Viability Assay

The 3-(4,5-dimethylthiazol-2-yl)-2,5-diphenyltetrazolium bromide (MTT) assay was firstly used to determine working concentration of kaempferol on cells. In short, HUVECs were seeded into each well of a sterile 96-well plate with density set at 5 × 10^3^/well. After HUVECs treated with drugs for 48 h, MTT solution with concentration set at 5 mg/ml was freshly prepared, and 10 μl of MTT solution was used for each well. The viability of cells was determined (Hu et al., 2019a; Hu et al., 2019b). An assay to determine the release of LDH was performed by applying a cytotoxicity detection kit plus (LDH) (Roche Diagnostics, Indianapolis, IN) with referring to the instructions with the kit. To measure the LDH content of each group, the formula as followed was used: Cytotoxicity (%) = (experimental value − low control)/(high control − low control) × 100.

### Endothelial Cell Migration Assay

To determine the migration ability of cells *in vitro*, a wound-healing assay was performed (Hu et al., 2019a; Hu et al., 2019b). Briefly, 20 × 10^4^ HUVECs in 1,000 μl of medium were plated onto each well of a sterile 12-well plate. After cells seeded for 24 h, with using a 200-μl pipette tip, a single wound was straightly made in the center of cell monolayer, and each well was rinsed with phosphate-buffered saline (PBS) which was pre-warmed to remove attached cells. Then, photos were captured under a phase-contrast microscope (A_t0_). The treatment of drugs lasted for 8 h and after drug treatment, three scratched areas in each well were selected freely and taken pictures (A_t8_). Finally, the wound area of endothelial cells before and after treatments of drugs was determined using Tscratch software (CSE lab, Switzerland). The recovery percentage of cells was quantized based on the formula as below: Recovery (%) = A_t0_ − A_t8_/A_t0_ × 100%.

### Tube Formation Assay

Tube formation assay was performed according to previous descriptions (Ashton et al., 1999; Hu et al., 2019a). Briefly, 24-well-plate was firstly covered by matrigel, and which was firstly allowed to polymerize for 1 h at 37 ˚C. Upon matrigel was polymerized, cell cultures treated by VEGF (5 ng/ml) or kaempferol were placed onto each well pre-treated with matrigel. The density of cells was 20 × 10^4^ cells/well. Medicine treatment was taken at 37°C and lasted for 8 h. After incubated with drugs, images of structures similar to tubes in each well were taken under a phase-contrast microscope. Pictures in control group and other groups treated by drugs were quantified according to the junction points. Branching points were counted manually.

### Zebrafish Angiogenesis Assay

Zebrafishes were fed in an environment supplied with flow water and regular aeration and the temperature was 28.5°C. The system was maintained at a cycle of 10 h: 14 h of dark/light. The experimental conduction on zebrafish was performed strictly following the requirements and guidelines of Animal and Plant Care Facility, approval by Animal Ethics Committee of HKUST. Following previous experience, healthy embryos were picked (Hu et al., 2019a; Hu et al., 2019b). Dechlorinated embryos were kept in a 12-well plate. After dechlorinated, 8~10 embryos were placed in each well and freely divided into different groups. Embryos were treated with water containing VEGF or kaempferol or their combination. After incubated with drugs for 48 h, to check viability, eyes and morphological characteristics of embryos were observed. Next, paraformaldehyde (4%) was used to fix the embryos at 4°C, and fixation reaction lasted for 20 h. Then the embryos were washed by PBS with 0.1% Tween-20 solution (PBST), 50% methanol and methanol. Each time lasted for 5 min. After rinsed, samples were kept in methanol and stored at −20°C. Then, by using PBST solution, embryos underwent rinsed continuously for another four times, again, each time lasted for 5 min. To begin with alkaline phosphatase-based vascular staining assay, samples were immersed in buffer 9.5T and then kept in nitro-blue tetrazolium/5-bromo-4-chloro-3-indolyl-phosphate (NBT/BCIP) (Cell Signalling Technology). To remove the interference of NBT/BCIP, after staining, the embryos were washed with PBST until the solution was clear. Finally, pictures representing sub-intestinal vessels were captured by a digital camera (Olympus DP71) under a stereomicroscope (Nikon AZ100). The quantification of the areas and branches vessels in sub-intestinal parts was performed by using an ImageJ software.

### Aortic Ring Sprouting Assay

The kaempferol-potentiated VEGF-triggered micro-vessel outgrowth was assessed by performing aortic ring sprouting experiments with minor modifications (Nicosia and Ottinetti, 1990). Briefly, thoracic aortas were gently dissected from Sprague-Dawley rats (6-week-old), and the tissue was washed by PBS which was pre-warmed. With application of small scissors, the fibroadipose tissue around the aorta was cut off, and the aortas were gently cut into pieces of fragments with thick set at 1-mm under a microscope. Then, the fragments were placed into a 24-well plate precoated with matrigel (200 μl). To cover aortic fragments, another 100 μl of matrigel was added into each well. After matrigel polymerized and solidified for 1 h, 500 μl medium containing VEGF and different concentrations of kaempferol were used treat the fragments. Avastin, at a concentration of 200 μg/ml and serving as a control, was used to treat aortic fragments. After medicine treatment lasted for 8 days, pictures of aortic fragments were taken under a phase-contrast microscope. An ImageJ software was used to perform the quantification of sprouting area of microvessels.

### Western Blot Analysis

Western blotting test was performed to determine the expression levels of some key proteins, including MMP-2, MMP-9 as well as phosphorylated p44/42 MAPK (Erk1/2), eNOS (S1177)VEGFR1 (Y103), and VEGFR2 (Tyr1175). Prior to drug treatment, basal medium without serum was used to treat cells for 1 h. After treatment of drugs, aspiration of medium was performed and subsequently, cultures were lysed in freshly made low-salt lysis buffer (2% SDS, 200 mM 2-mercaptoethanol, 10% glycerol, 125 mM Tris–HCl, pH 6.8), and the samples were transferred into centrifuge tubes. The cell lysates in tubes were boiled at 95°C for three times, each for 5 min. The protein extracts were analyzed and separated by applying 7% or 8% acrylamide gel electrophoresis. Protein transferring reaction was taken at 4°C and nitrocellulose membranes was used here. Then, blocking reaction was taken for 60 min at room temperature by using 5% milk, which was prepared in a Tris-buffered saline containing 0.1% TBST. The membranes were immersed in primary antibody at 4°C. All primary antibodies used were diluted 1,000×, and the membranes were incubated with primary antibodies for 24 h. After incubated with primary antibody, membranes were washed and then subjected to react with horseradish peroxidase-conjugated secondary anti-rabbit antibody. Here, the secondary antibody was diluted at 1:2,000. After reacted with secondary antibody for 2 h, membranes were rinsed by TBST solution for four times, and each time lasted for 5 min. By dropping ECL (Invitrogen), the reactive bands in membrane were visualized. Pictures representing reactive bands were obtained by using Chemidoc Imaging System (Bio-Rad; Hercules, CA). The band intensities of control group and drug-treated groups, run on the same piece of gel and taken pictures under standardized ECL conditions, were analyzed and went on comparison by using the related software, which were performed based on a calibration plot from a parallel gel with one of the samples, diluted at a series of different ratios. To quantify Western blot in phosphorylation in each group, the band at 10 min for each group was compared with the band at 0 min after comparison with its corresponding total protein. Then, each group was compared with the control group.

### Fluorescence Intensity Analysis

HUVEC cells (20 × 10^4^) were plated into each well of a sterile 12-well plate. After cultured for 24 h, fresh medium supplemented with 5 ng/ml biotinylated VEGF, or together with kaempferol was used to treat cells. The group incubated with fresh medium only served as a background control. Drug treatment lasted for 24 h at 37°C, Then, to hybridize with the biotinylated VEGF, Streptavidin-Cy5™ was added into cultures, and the hybridization reaction lasted for 60 min at 37°C. After rinsed, cultures were under fixation in ethanol, and images were captured under a laser confocal fluorescent microscopy. The fluorescence intensity of each photo was quantified with application of relevant software.

### Skin Cell Migration Assay

A cell migration assay was used to determine wound recovery of skin cell *in vitro* by using HaCaT cells. Briefly, 50 × 10^4^ HaCaT cells were seeded into each well of a sterile 6-well plate. After cells allowed to grow to a confluent monolayer, a scrape was performed in the middle of each well with application of a sterile P200 micropipette tip. At different time of drug treatment (0 and 16 h), pictures of wound area in each well were taken by using a phase-contrast microscope with randomly determined six points per well. The wound area was then analyzed with application of Image J software. The relative wound area was obtained by calculation of dividing the change in the wound area of drug-treated group by that of the control group without drug treatment in each experiment.

### Monocyte Cell Migration Assay

A cell migration assay was performed to determine wound recovery of monocyte cell *in vitro* by using murine RAW 264.7 macrophages. Briefly, 60 × 10^4^ macrophages were seeded into each well of a 12-well plate. After cells growing to a 90% confluence, a scrape was performed in the center of the monolayer in each well by using a sterile P200 micropipette tip. At different time of drug treatment (0 and 24 h), pictures of wound area in each well were taken by using a phase-contrast microscope with randomly determined six points per well. The number of cells located within the wound area was used to quantify cell migration and analyzed with application of Image J software. The relative cell number was obtained by calculation of dividing the change in the cell number of drug-treated group by that of the control group without drug treatment in each experiment.

### Statistical Analysis

Protein was determined by Bradford method (Hercules, CA). Statistical analysis was performed using one-way analysis of variance (ANOVA) followed by a Bonferroni multiple comparisons test using the SPSS 16.0 software. Data were shown as the mean ± standard error of the mean (SEM). The statistical significance was set at *p* < 0.05.

## Results

### Kaempferol Binds VEGF

**Figure 1:**
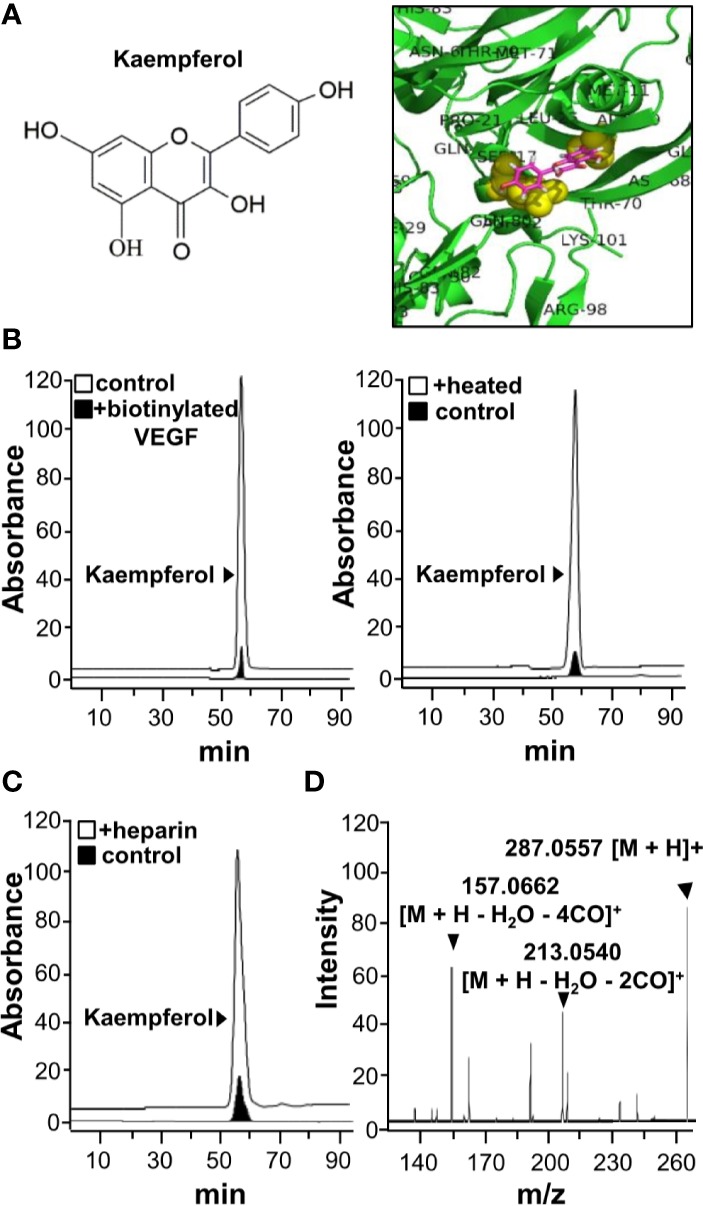
Kaempferol binds with VEGF. **(A)** Structure of kaempferol (left) was shown, and protein structure of VEGF (i.e. VEGF-A) was downloaded from PDB for performing molecular auto-docking. Visualization of the binding interaction of kaempferol-VEGF was shown (right). VEGF: green; kaempferol: sticks, color of carbon: pink, oxygen: red, hydrogen: silver; the proposed binding site: yellow. **(B)** UPLC chromatogram was used to detect the amount of kaempferol in supernatant after biotinylated VEGF or VEGF (66.1 ng/ml) in an immunoprecipitation assay by streptavidin magnetic beads (left panel). The heated denature VEGF was tested for binding to kaempferol (right panel). **(C)** The binding of VEGF with kaempferol was interfered in the present of heparin (500 μg/ml; left panel). **(D)** Having an electrospray ionization (ESI) source with ionization set at the positive mode of a mass spectrometer, the mass-charge ratio of kaempferol was determined (right panel). The typical figure was demonstrated, *n* = 3. VEGF, vascular endothelial growth factor.

Kaempferol was identified to bind VEGF from our HerboChips screening. The chemical structure of kaempferol was shown (**Figure 1A**). Molecular docking was performed with PyMOL molecular graphic system and AutoDock tool. The proposed binding interaction between kaempferol and VEGF protein was shown (**Figure 1A**). The binding affinity between kaempferol and VEGF protein was estimated from −6.9 to −6.1. According to auto-docking result, the specific binding site of kaempferol to VEGF protein was shown to be located at heparin binding domain of the growth factor. This binding region is in contrast to that of polydatin or resveratrol (Hu et al., 2019a; Hu et al., 2019b). To confirm the binding of kaempferol to VEGF, an immuno-precipitation assay was applied here. The precipitated kaempferol in the sample having biotinylated VEGF was much higher than that deriving from without VEGF or with heated denatured VEGF (**Figure 1B**), suggesting the binding specificity. Moreover, the binding of kaempferol to VEGF was markedly displaced by heparin (**Figure 1C**). The precipitated kaempferol was further analyzed by LC-MS/MS analysis. The [M+H]^+^ ion in positive mode at m/z 287.0557 was identified for kaempferol, and which produced smaller fragments of m/z 213.0540 and 157.0662 (**Figure 1D**). Therefore, the aforementioned results gave support to a direct binding interaction between VEGF protein and kaempferol.

### Kaempferol Potentiates VEGF-Mediated Angiogenesis

**Figure 2:**
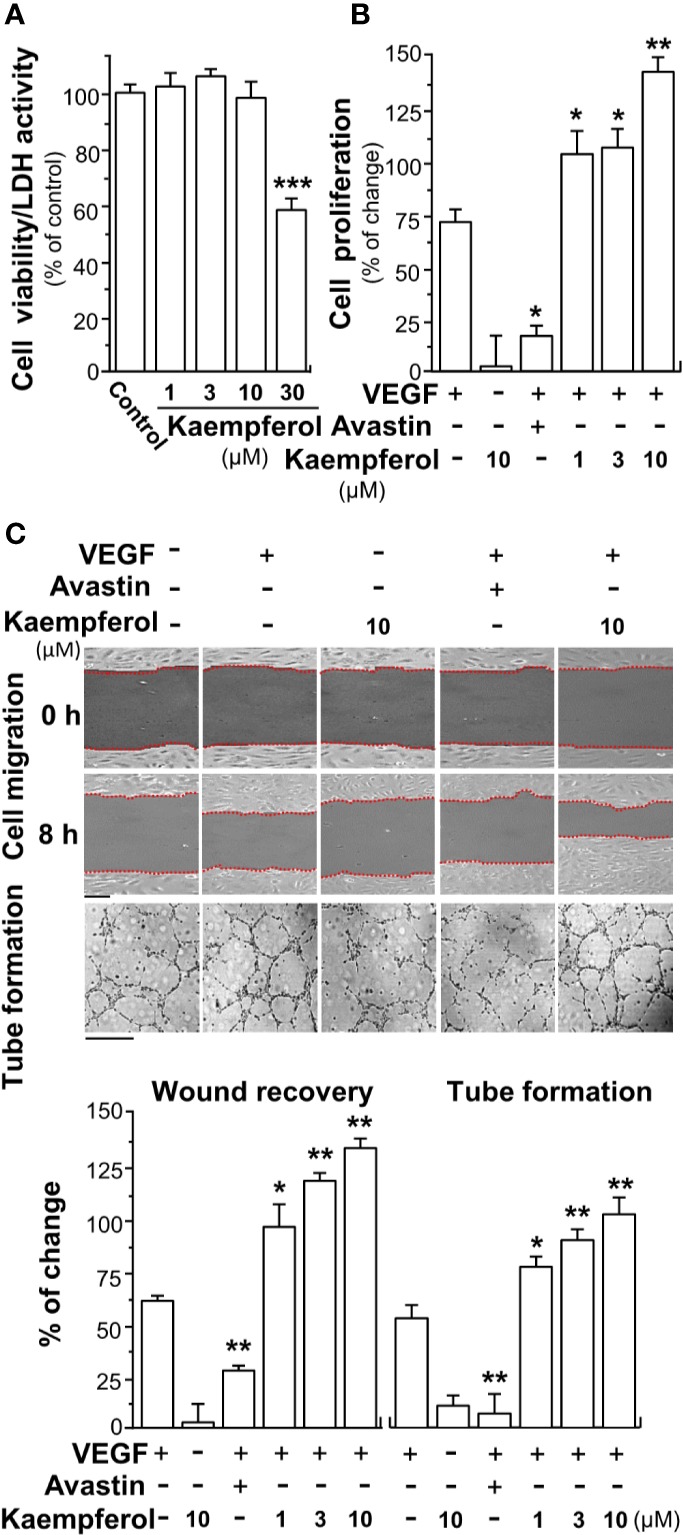
Kaempferol promotes VEGF-mediated endothelial cell proliferation, cell migration, and tube formation. **(A)** Different concentrations of kaempferol were applied onto endothelial cells for 48 h, and MTT assay and LDH cytotoxicity assay were determined. **(B)** HUVECs were seeded onto a 96-well plate at 5,000 cells/well and treated with kaempferol for 48 h with or without VEGF. **(C)** In cell migration assay, HUVECs at 20 × 10^4^ cells/well were plated onto a 12-well plate. A wounded endothelial cell monolayer was created manually at the bottom of wells, and images representing recovery of wounds were taken at 0 and 8 h, separately, by a phase-contrast microscope. HUVECs were incubated with VEGF, with or without kaempferol or Avastin. In tube formation assay, endothelial cells were seeded onto a 12-well plate at 20 × 10^4^ per well. The well was pre-coated with matrigel. VEGF, together with or without kaempferol, was applied to treat cells for 8 h. To quantify the images of tube formation, three fields in one photo were randomly selected, and branching points were determined manually. In all experiments, VEGF was at a concentration of 5 ng/ml, and Avastin (a positive control) was set at 200 μg/ml. Data are demonstrated as mean ± SEM of the percentage of change as compared to control group, where *n* = 4; *p* < 0.05 *p < 0.01 **p < 0.001 (***) vs VEGF-treated group. Bar = 40 μm. MTT, 3-(4,5-dimethylthiazol-2-yl)-2,5-diphenyltetrazolium bromide; HUVEC, human umbilical vein endothelial cell.

Based on the binding between kaempferol and VEGF, we suspected that kaempferol might exert effects in VEGF-induced angiogenesis and cell proliferation. Having kaempferol up to 10 µM, the treatment on endothelial cells exerted no significant effects on cell viability (**Figure 2A**). The proliferation of endothelial cell was triggered by application of VEGF in a dose-dependent manner (**Supplementary Figure 1**), and which indicated that 5 ng/ml VEGF at the sub-maximum response could be used for the assay here. The cell proliferation, triggered by 5 ng/ml VEGF, was further potentiated, in a dose-dependent manner, in the presence of kaempferol (**Figure 2B**). Avastin, a monoclonal antibody against VEGF, served as a control to suppress VEGF effect (Michael, 2014; Hu et al., 2019a; Hu et al., 2019b).

Endothelial cell migration and tube formation contribute to new blood vessel formation, and they are initiating the repairing of injured vessel. Here, the promoting effect of kaempferol on the migration of endothelial cells was determined by wound healing assay *in vitro*. The wound cultures were treated by VEGF, or together with kaempferol. As a control, the endothelial cell migration, induced by VEGF, was decreased in the present of Avastin (**Figure 2C**). Kaempferol alone exerted no effect on cell migration. The treatment of kaempferol and VEGF together in HUVECs significantly reduced the denuded area, i.e. increase wound recovery (**Figure 2C**). To further study the enhancing effects of kaempferol in angiogenesis, the tube formation was determined in VEGF-treated endothelial cells. After treated with VEGF, elongated and solid tube-like structures were formed. An interruption of capillary-like tubes was obviously revealed after Avastin treatment; however, the connections in capillary-like tubes were robustly increased after co-treatment of VEGF with kaempferol (**Figure 2C**). Kaempferol alone exerted no effect on tube formation. These pharmaceutical investigations contributed a lot to a conclusion that kaempferol could potentiate the VEGF-induced angiogenesis in cultured endothelial cells.

**Figure 3:**
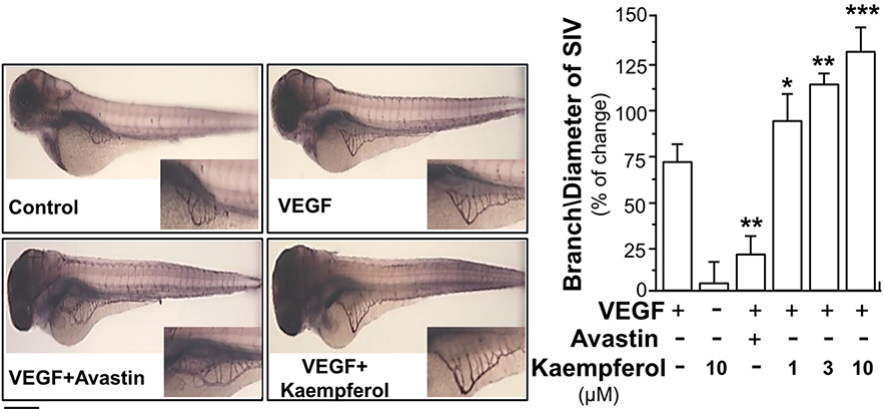
Kaempferol potentiates angiogenesis *in vivo*. Healthy zebrafish embryos were picked out for each group and placed onto a 12-well plate with density set at 8–10 embryos/well. Embryos were fed with PU water containing 5 ng/ml VEGF with or without kaempferol or 200 μg/ml Avastin on 1^st^ day of development. After drug treatment for 48 h, the fish embryos were stained. The blood vessels located in the sub-intestinal were captured for blood vessel formation. Images represented the basket of sub-intestinal vessels of the zebrafish embryos developed for 3 days. The branches and area of sub-intestinal vessels in control group and medicine-treated group were quantified with application of Image J software. Results are shown as the percentage of change as compared to control (no drug) in mean ± SEM, where *n* = 3; *p* < 0.05 *p < 0.01 **p < 0.001 (***) vs VEGF-treated group. Bar = 40 μm.

In zebrafish model, the neo-vascularization of sub-intestinal vessels was used to investigate the effect of kaempferol in VEGF-mediated angiogenesis *in vivo*. Zebrafish embryos were dechlorinated and subsequently incubated with VEGF, Avastin, or kaempferol. After treatment of VEGF, the blood microvessels in sub-intestinal part were elongated; besides, the area was broadened (**Figure 3**). There were few changes at sub-intestinal vessels in treatment of kaempferol alone. However, kaempferol applied together with VEGF could enhance the VEGF-mediated vessel formation of the embryos in a concentration-dependent manner (**Figure 3**). As expected, Avastin suppressed the VEGF-induced vessel formation.

**Figure 4:**
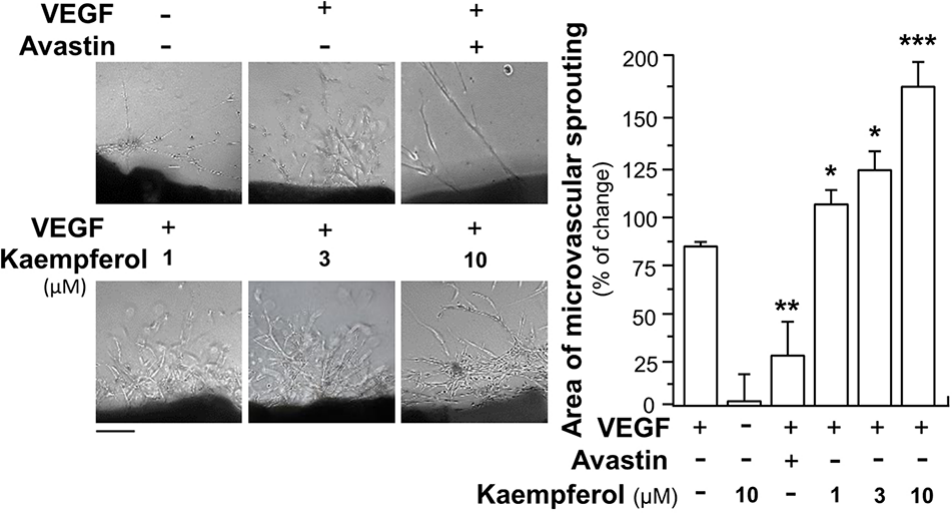
Kaempferol potentiates angiogenesis *ex vivo* microvascular sprouting. Thoracic aortas were removed from 6-week-old Sprague-Dawley rats, and aortic ring fragments (cut into 1-mm thick) were cultured in matrigel and incubated with 5 ng/ml VEGF with or without kaempferol or 200 μg/ml Avastin. After drug treatment for 8 days, micovessels in each piece of fragment were captured for microvascular outgrowth. Images represented the microvascular outgrowth, and the area of microvascular sprouting in the control group and drug-treated group were quantified with application of Image J software. Results are shown as the percentage of change as compared to control (no drug) in mean ± SEM, where *n* = 3; *p* < 0.05 *p < 0.01 **p < 0.001 (***) vs VEGF-treated group. Bar = 40 μm.

The potentiating activities of kaempferol on VEGF-triggered angiogenic functions were also proved by performing an *ex vivo* aortic ring sprouting assay. Rat aortic fragments were cultured on the matrigel and treated with drugs, including VEGF, Avastin or kaempferol. Section of aortic fragments was determined, and the angiogenic responses were monitored for eight days. The microvascular sprouting was visibly formed with the VEGF application; while fewer amounts of micro-vessels were formed in the group treated by Avastin (**Figure 4**). Kaempferol alone did not alter changes of aortic fragments. With co-applied VEGF, kaempferol prominently promoted the VEGF-induced neovascularization in aortic fragments in a concentration-dependent manner, i.e. promoting the outgrowth of microvascular outgrowth (**Figure 4**).

### Kaempferol Enhances VEGF-Induced Signaling

**Figure 5:**
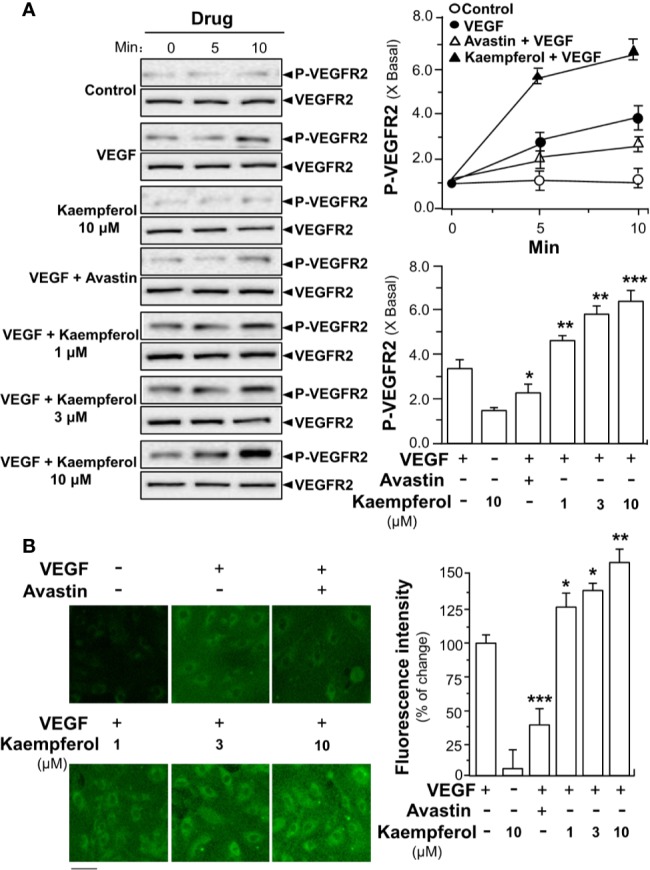
Kaempferol enhances VEGF binding to VEGFR2 and its phosphorylation. **(A)** Endothelial cells were seeded onto each well of a 12-well plate with the density set at 20 × 10^4^ per well. VEGF (5 ng/ml), was used in presence or absence of kaempferol or Avastin (200 μg/ml). The cell lysates were collected at different time points. Western blotting (left panel) was used to probe phosphorylated and total expressions of VEGFR2. Quantitation was done at different time, as well as different drug treatments, at 10 min of activation (right panel). **(B)** Fluoresence-labeled VEGF (5 ng/ml) was applied onto HUVECs, with or without kaempferol, for 24 h. The fluorescence intensity in fluoresence-labeled VEGF minus background (no drug) was considered as 100%. Avastin (200 μg/ml) served as a control. Right panel is the quantitation values deriving from left panel of confocal micrograph. Data are indicated as X Basal setting as 1, or the percentage of change as compared to biotinylated VEGF group, mean ± SEM, where *n* = 3–4; *p* < 0.05 *p < 0.01 **p < 0.001 (***) vs VEGF-treated group. Bar = 40 μm.

To demonstrate the role of kaempferol in potentiating VEGF-triggered angiogenic signaling mechanisms in endothelial cells, the expressions of phosphorylated VEGFR2, eNOS and Erk were determined here. After treated with VEGF, the expression levels of phosphorylated VEGFR2, an active form of VEGFR2, and total VEGFR2 were determined by Western blotting (**Figure 5A**). Comparing with the control group, VEGF treatment visibly enhanced the expressions of phosphorylated NOS in a time-dependent manner. The maximal activation was ~4-fold at 10 min (**Figure 5A**). Meanwhile, application of Avastin in endothelial cells markedly suppressed the VEGF-mediated phosphorylation of VEGFR2 in both time- and dose-dependent manners without altering the total expression of VEGFR2 protein. In cultured endothelial cells, kaempferol alone exerted no effect on VEGFR2 phosphorylation. The kaempferol-treated endothelial cells obviously potentiated the VEGF-triggered the expression of phosphorylated VEGFR2 in time- and dose-dependent manners: the maximal increase could be achieved at ~6-fold (**Figure 5A**). Kaempferol binding to VEGF heparin binding domain was expected to increase the binding interaction of VEGF to its receptor VEGFR2, potentiating the receptor activation. To confirm this possibility, the binding activity of VEGF onto cell cultures was measured by using a fluorescence-labeled VEGF. Application of kaempferol increased the binding activity of VEGF to cells: this binding enhancement, triggered by kaempferol, was in a dose-dependent manner (**Figure 5B**).

**Figure 6:**
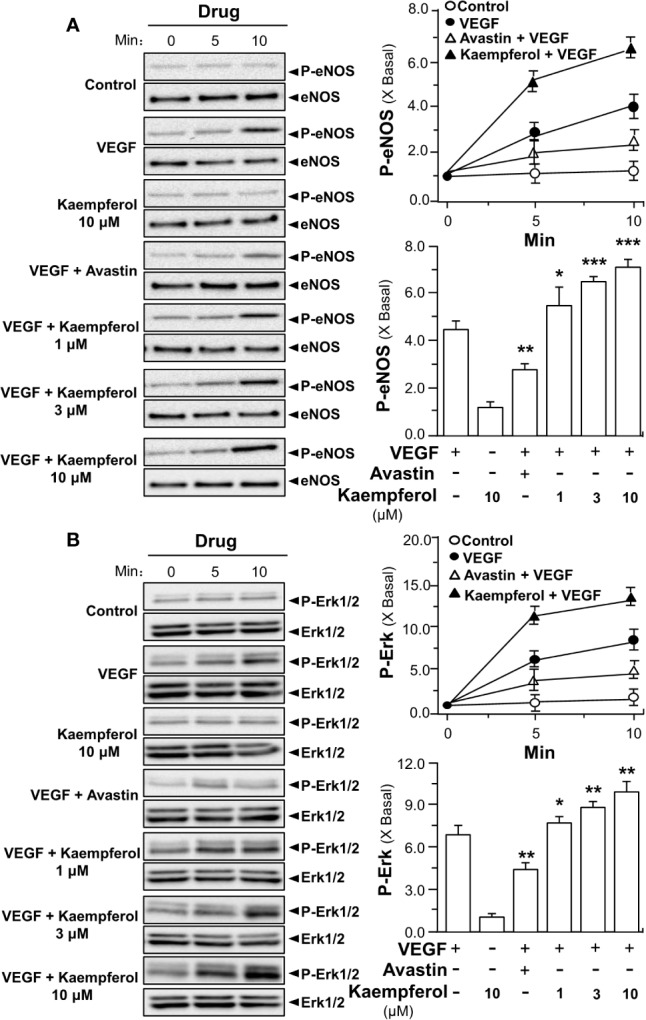
Kaempferol potentiates VEGF-triggered phosphorylations of eNOS and Erk. 20 × 10^4^ HUVECs were seeded into each well of a 12-well plate. VEGF (5 ng/ml) was determined with or without kaempferol or Avastin (200 μg/ml). The lysates of endothelial cells were collected at different time intervals, as demonstrated. Phosphorylated and total proteins of eNOS **(A)** and Erk **(B)** were separately analyzed by Western blotting. Right panel is the quantitation values deriving from left panel. Quantitation was done at different time, as well as different drug treatments, at 10 min of activation. Results are expressed as the fold of change as compared to control (X Basal), where the control group (no drug) was set as 1, mean ± SEM, where *n* = 3; *p* < 0.05 *p < 0.01 **p < 0.001 (***) vs VEGF-treated group. eNOS, endothelial nitric oxide synthase; Erk, extracellular signal-regulated kinase.

In parallel, the applied VEGF increased the downstream mechanism signaling mediated by VEGFR2. The VEGF-induced expression levels of phosphorylated eNOS and Erk were potentiated by fivefold to ninefold in the co-treatment of kaempferol: the protein expression of total eNOS and Erk remained unchanged (**Figures 6A, B**). Avastin showed contrary effects in VEGF-mediated phosphorylations of eNOS and Erk, acting as a control (**Figures 6A, B**). Kaempferol alone did not alter the expressions of phosphorylated eNOS and Erk. These results suggested the kaempferol-bound VEGF might have stronger activation to its receptor.

**Figure 7:**
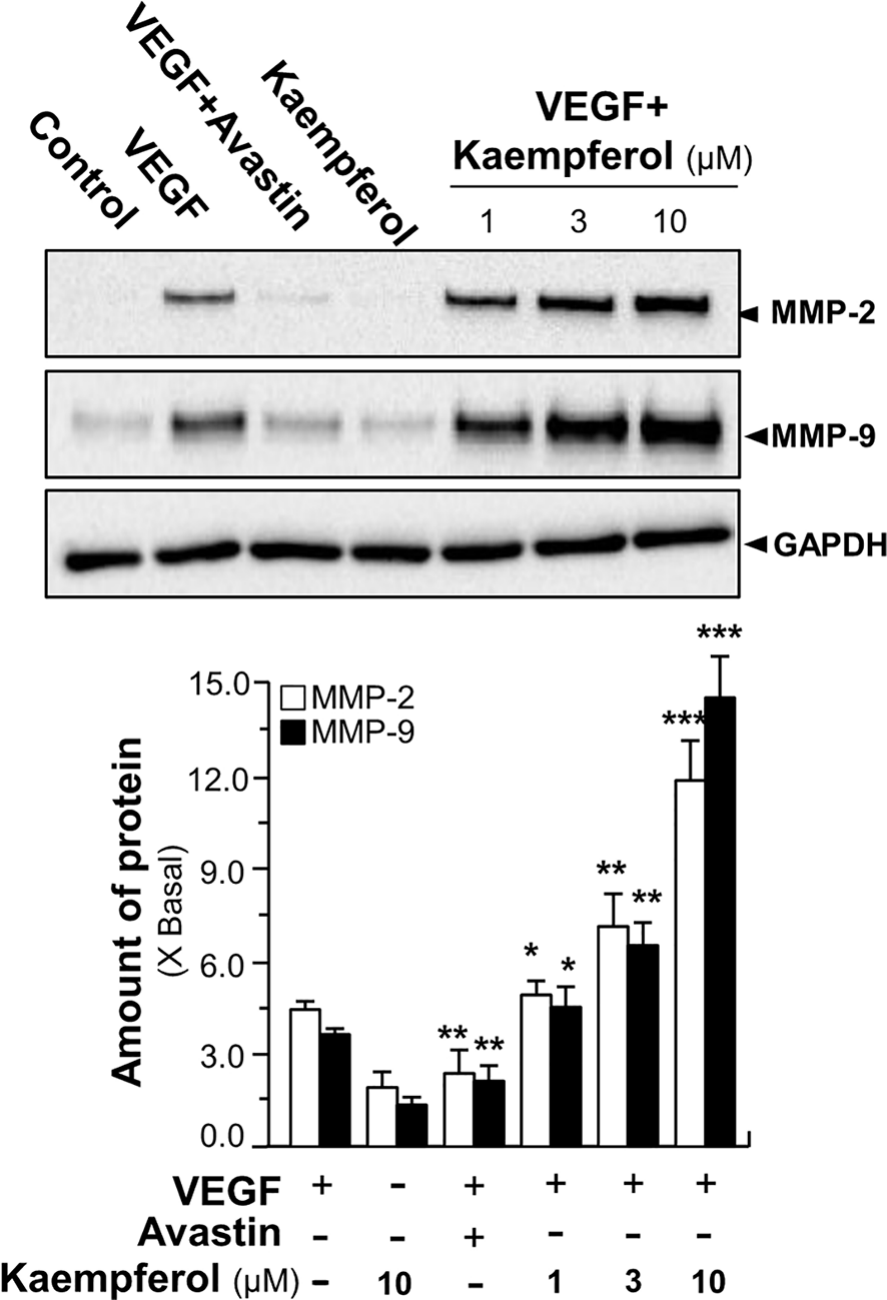
Kaempferol enhances VEGF-triggered expressions of MMP-2 and MMP-9. HUVECs, at a density of 40 × 10^4^ per well, were seeded in a six-well plate. VEGF (5 ng/ml) was applied with or without Avastin or kaempferol at different concentrations. The cell lysates were collected after drug treatment. The protein expressions of MMP-2 and MMP-9 were determined by performing Western blotting (upper panel). GAPDH served as a loading control. Quantitation was done from the band intensity in Western blotting (lower panel). Results are expressed as the fold of change as compared to control (X Basal), where the control group (no drug) was set as 1, mean ± SEM, where *n* = 3; *p* < 0.05 *p < 0.01 **p < 0.001 (***) vs VEGF-treated group. MMP, matrix metalloproteinase; GAPDH, glyceraldehyde 3-phosphate dehydrogenase.

We further investigated the signaling mechanism of kaempferol in angiogenic function by determining expressions of factors playing key roles in cell migration and invasion (Moses, 1997). Thus, the expressions of MMP-2 and MMP-9 were analyzed by performing Western blotting assay in endothelial cells treated by VEGF with or without kaempferol. Application of VEGF remarkedly increased the expression levels of MMP-2 and MMP-9 by ~5-fold and ~3-fold, respectively (**Figure 7**). At the same time, Avastin treatment attenuated the VEGF-mediated expressions. In cultured endothelial cells, kaempferol application alone showed no effect on expression levels of MMP-2 and MMP-9; while the co-treatment of kaempferol and VEGF induced expression levels of MMP-2 and MMP-9 proteins at ~9-fold and ~10-fold, respectively (**Figure 7**).

### Kaempferol Potentiates VEGF-Triggered Functions

**Figure 8:**
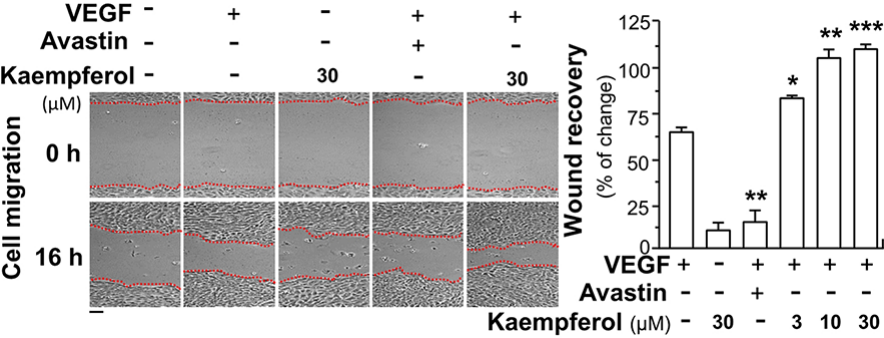
Kaempferol enhances VEGF-triggered migration of HaCaT cells. Keratocytes (HaCaT cells) at 50 × 10^4^ cells/well were plated onto a six-well plate. A wounded skin cell monolayer was created manually at the bottom of wells, and pictures representing wound recovery were captured at 0 and 16 h, separately, by a phase-contrast microscope. HaCaT cells were incubated with VEGF, with or without kaempferol or Avastin. Data are demonstrated as mean ± SEM of the percentage of change as compared to control group, where *n* = 3; *p* < 0.05 *p < 0.01 **p < 0.001 (***) vs VEGF-treated group. Bar = 40 μm.

Besides angiogenesis, we aimed to reveal the outcome of kapempferol binding to VEGF in affecting other biological activities of VEGF, at least in terms of activation of other forms of VEGF receptor. The moving ability of epidermal keratinocytes contributes to a high degree of wound re-epithelialization. Here, we investigated the VEGF-mediated wound healing process in cultured HaCaT cells. In a scratch wound-healing assay, the cells were incubated with VEGF, or kaempferol, and the cell migration across the wound space was analyzed after 16 h. Avastin exerted inhibitory effects on cell migration (**Figure 8**). In comparison with control group, the treatment of HaCaT cells with kaempferol and VEGF together significantly enhanced the cells migrating across the denuded space in a dose-dependent manner, which provided evidence to positive role of kaempferol in promoting VEGF-mediated migration of keratinocyte (**Figure 8**).

**Figure 9:**
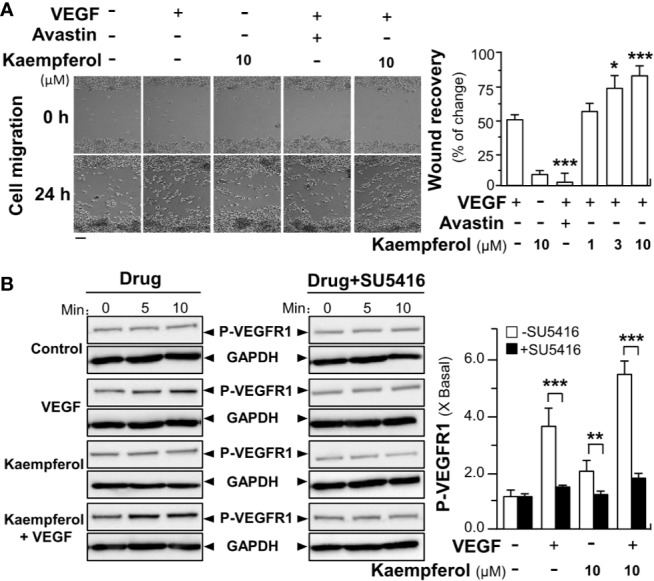
Kaempferol promotes VEGF-induced migration of macrophages *via* VEGFR1 phosphorylation. **(A)** RAW264.7 cells at 60 × 10^4^ cells/well were seed onto a 12-well plate. A wounded monocyte cell monolayer was created manually at the center of wells, and images representing wound recovery were captured at 0 and 24 h, separately, under a phase-contrast microscope. Macrophages were incubated with VEGF, with or without kaempferol. The recovery of wound is measured by the cell occupant per unit area. Data are demonstrated as mean ± SEM of the percentage of change as compared to control group. Bar = 100 μm. **(B)** Macrophages were plated into a 12-well plate at 60 × 10^4^ cells per well. The cells were treated with VEGF (5 ng/ml) with or without kaempferol. An inhibitor for VEGFR1 (SU5416 at 50 μM) was used. Data are expressed as X Basal, where the control was set as 1. All data are in mean ± SEM, where *n* = 3; *p* < 0.05 8*p* < 0.01 **p < 0.001 (***) vs VEGF-treated group or indicated.

The VEGFR1 mRNA is being expressed in human peripheral blood monocytes, and VEGFR1 tyrosine kinase contributes greatly to VEGF-mediated cell migration of macrophages (Barleon et al., 1996). Here, the role of kaempferol in VEGF-induced wound healing process and phosphorylations of VEGFR1 in cultured macrophage cells was tested. In a cell migration assay, macrophages were incubated with VEGF, or kaempferol, and the cell migration across the wound space was analyzed after 24 h. Avastin demonstrated inhibitory effects on cell migration (**Figure 9A**). The treatment of macrophages with kaempferol and VEGF together obviously increased the cell migrating across the denuded space in a dose-dependent manner, supporting a positive role of kaempferol in migration of monocytes (**Figure 9A**). Besides, the effects of kaempferol on VEGF-mediated phosphorylations of VEGFR1 in macrophages were further tested by western blotting analysis. Application of VEGF markedly increased the phosphorylations of VEGFR1 by ~4-fold (**Figure 9B**). Meanwhile, kaempferol treatment alone demonstrated little effects on VEGFR1 phosphorylation; while the kaempferol-treated macrophages significantly up regulated the VEGF-induced VEGFR1 phosphorylation at ~6-fold, respectively (**Figure 9B**). Moreover, the treatments of SU5416 further blocked VEGF/kaempferol-induced phosphorylation, which almost restored back to the control level. Thus, it could be concluded that the signaling, induced by kaempferol, was similar to the selective VEGFR1 kinase inhibitor.

## Discussion

TCM is a great source to find possible treatment of various diseases, and additionally there is a steady growth about the application of botanical dietary supplements. In our HerboChips drug screening, Ginkgo Folium was identified here by using VEGF as a target protein. Kaempferol, an abundant active flavonoid in Ginkgo Folium, was shown to bind VEGF, and subsequently which potentiated angiogenic effects of VEGF. The extract of Ginkgo Folium is one of the top 10 commonly used herbal products (Leung, 1982). Pharmacological activities of the leaves of *Ginkgo biloba* are proposed to have cardiac protection, stimulating blood flow, radical scavenging and enhancing activities of anti-platelet activating factors (Beek, 2000). Active constituents, e.g. bilobalide, ginkgolides and flavonoids, in Ginkgo Folium play functions in antioxidant (Chen et al., 1999), anti-ischemic (Chung and Harris, 1999) and anti-convulsant (Sasaki and Hatta, 1999). Kaempferol is not only found in Gingko Folium; this flavonoid is commonly found in many edible plants, e.g. apples, strawberries and beans. Kaempferol has been reported to have anti-apoptosis (Choi and Ahn, 2008), anti-inflammation (Bobe et al., 2010), anti-oxidation (Tatsimo et al., 2012), and anti-carcinogenic effects (Gates et al., 2009). However, there are very few investigations targeting the potentiating effects of kaempferol in angiogenesis. Petpiroon et al. (2015) demonstrated that kaempferol could promote wound healing using a human keratinocyte model, which suggested the potentiating effect of kaempferol in angiogenesis. Angiogenesis contributes to pathologic processes in many diseases, including diabetes, ischemic heart disease, cancer, and chronic inflammation (Carmeliet, 2003). Here, we highlighted the pharmacological activities of kaempferol in VEGF-induced angiogenesis. Application of kaempferol in cultured HUVECs could significantly promote VEGF-mediated endothelial cell proliferation, cell migration and tube formation; and in *in vivo* the effects of kaempferol in angiogenesis were demonstrated in vessel formation of zebrafish embryos and micro-vessels outgrowth from aortic fragments. In addition, we illustrated the underlying mechanism signaling by which kaempferol positively controls the VEGF-triggered expressions of phosphorylated VEGFR2, eNOS, and Erk. Thus, the angiogenic effect of kaempferol is proposed to be initiated by its binding with VEGF, which therefore potentiates the activation of VEGF on its receptor. Besides, MMPs are known to play critical roles in initiation of angiogenesis, which contribute to re-organization of extracellular matrix, break down stroma and extracellular matrix proteins, and promote endothelial cells moving toward sources of growth factors (Herron et al., 1986; Liotta, 1986). The expressions of MMP-2 and MMP-9 were measured in VEGF-induced cultures, and which was potentiated by kaempferol.

In natural products, kaempferol has chemical analogs sharing a close structure similarity, e.g. quercetin and kaempferol-3-O-rutinoside. The difference between kaempferol and quercetin in pharmacological properties did exist in regulating pro-inflammatory genes (Crespo et al., 2008) and in interacting with immunoglobulin (Liu et al., 2008). Inspired by these studies, we have search possible interactions of VEGF with kaempferol analogs. According to the molecular docking analysis, quercetin and kaempferol-3-O-rutinoside could possibly bind to VEGF. In line to this notion, kaempferol-3-O-rutinoside has been shown to promote keratinocyte migration through FAK and Rac1 activation (Petpiroon et al., 2015).

Different growth factors, e.g. EGF, bFGF, VEGF, and PDGF, and their corresponding receptors are known to play key roles in angiogenic regulation. Among these growth factors, VEGF is considered as the most important one. VEGF reacts with two main VEGF receptors, namely VEGFR1 and VEGFR2, and VEGFR2 has been proved to be responsible for the majority of VEGF-mediated angiogenic activities. Once interacted with VEGFR2, VEGF takes the main responsibilities to activate downstream signaling, including eNOS and Erk, as to enhance angiogenic process. HerboChips screening is tailored to find compounds from natural source that bind to growth factors (Lee et al., 2016). In previous reports, polydatin and its closely related analogs, resveratrol, were shown to bind with VEGF, and this binding, similar to Avastin, inhibited angiogenic functions of VEGF (Hu et al., 2019a; Hu et al., 2019b). The inhibitory effects of polydatin/resveratrol in VEGF-treated cultures could be attributed to its occupation of possible binding sites between VEGF and VEGFR, which subsequently decreased the binding of VEGF to VEGFR. However, kaempferol is different to that of polydatin/resveratrol. The binding of kaempferol enhanced the VEGF-triggered phosphorylations of VEGFR2, eNOS, and Erk. The potentiating activity of kaempferol might be accounted by its direct binding with VEGF at the heparin binding domain, i.e. to increase the binding of VEGF to its receptor, which could be different from binding sites of polydatin/resveratrol to VEGF. Thus, kaempferol at low concentration could be used for treatment of ischemic heart disease related with angiogenesis, including coronary heart disease and myocardial infarction. Contrary, studies have proposed kaempferol could suppress angiogenesis through inhibiting VEGFR2 expression (Luo et al., 2012; Liang et al., 2015). However, the authors did not identify the interacting target of VEGF or VEGF receptor. Besides, a rather high concentration of kaempferol (i.e. 80 µM) was applied in their reports: this concentration of kaempferol showed cell death activity, as shown in our study, as well as in other (Liang et al., 2015).

The important roles of epidermal cell migration in wound healing have been reported (Gao et al., 2010; Walter et al., 2010). Indeed, the migration of keratinocyte toward the wound site is a step of great significance in restoring a barrier in cutaneous wounds (Santoro and Gaudino, 2005; Ranzato et al., 2008). Herein, we demonstrated, for the first time, that kaempferol has a positive effect on VEGF-mediated migration of human keratinocyte cells, which was consistent with our findings obtained based on endothelial cells. Because the migration of keratinocyte plays critical roles in the wound re-epithelialization, the information obtained from here should support development of kaempferol for clinical application as an active agent for wound healing and related applications.

VEGFR1 is being expressed in monocytes, and it is highly expressed during the differentiation of monocyte–macrophage lineage (Sawano et al., 2001). VEGFR1 promotes migration of macrophage through PI3K and PLCγ signaling pathways (Weddell et al., 2018). Here, we demonstrated that kaempferol could enhance VEGF-mediated migration of macrophage cells by enhancing VEGF-induced VEGFR1 phosphorylations. Because pro-inflammatory mediators are mainly produced by macrophages, and thus macrophage migration from circulation to injured tissues plays a vital role in wound healing. Polarization of macrophages is relating to the remodeling of homeostatic tissue, inflammation resolution and tissue repair (Mantovani et al., 2013). Thus, our investigations may shed light on the positive effects of kaempferol on macrophage migration, and tissue regeneration *via* modulation of macrophage through VEGFR1 signaling pathway.

## Data Availability Statement

All datasets generated for this study are included in the article/**Supplementary Material**.

## Ethics Statement

The animal study was reviewed and approved by Animal Ethics Committee of HKUST.

## Author Contributions

W-HH wrote the paper and performed most of the experiments. KT revised the paper, conceived, and designed the research. H-YW took part in most of the experiments. Y-TX and KD helped to make gels for western blot assay and YT conducted HPLC. RD and Q-PX performed data analysis for the cell migration assay. GC helped to do cell culture. TD provided reagents and Q-WQ took part in screening of HerboChips analysis.

## Funding

This work is supported by Shenzhen Science and Technology Innovation Committee (ZDSYS201707281432317; JCYJ20170413173747440; JCYJ20180306174903174), China Post-doctoral Science Foundation (2019M653087), Zhongshan Municipal Bureau of Science and Technology (ZSST20SC03), Guangzhou Science and Technology Committee Research Grant (GZSTI16SC02; GZSTI17SC02), Hong Kong RGC Theme-based Research Scheme (T13-607/12R), Hong Kong Innovation Technology Fund (UIM/340, UIM/385, ITS/500/18FP; TCPD/17-9), TUYF19SC02, PD18SC01, and HMRF18SC06.

## Conflict of Interest

The authors declare that the research was conducted in the absence of any commercial or financial relationships that could be construed as a potential conflict of interest.

## Supplementary Material

Supplementary Figure 1 VEGF promotes endothelial cell proliferation in a concentration-dependent manner. HUVECs were seeded onto a 96-well plate at 5,000 cells/well and treated with or without VEGF at different concentrations. The cell viability was assayed by MTT after 48 h of treatment. Data are demonstrated as Mean ± SEM of the percentage of change as compared to control group (no VEGF), where *n* = 5.

## Abbreviation

MMP-2, matrix metalloproteinase-2; MMP-9, matrix metalloproteinase-P; Erk, extracellular signal-regulated kinase; eNOS, endothelial nitric oxide synthase; GAPDH, glyceraldehyde 3-phosphate dehydrogenase; VEGF, vascular endothelial growth factor; VEGFR1, VEGF receptor 1; VEGFR2, VEGF receptor 2; VEGFR3, VEGF receptor 3; HUVECs, human umbilical vein endothelial cells.
