# Supplementary figures and images for: Kaempferol, a Major Flavonoid in Ginkgo Folium, Potentiates Angiogenic Functions in Cultured Endothelial Cells by Binding to Vascular Endothelial Growth Factor
Source: Front Pharmacol. 2020 Apr 28;11:526. doi: 10.3389/fphar.2020.00526 (PMC7198864; doi:10.3389/fphar.2020.00526)

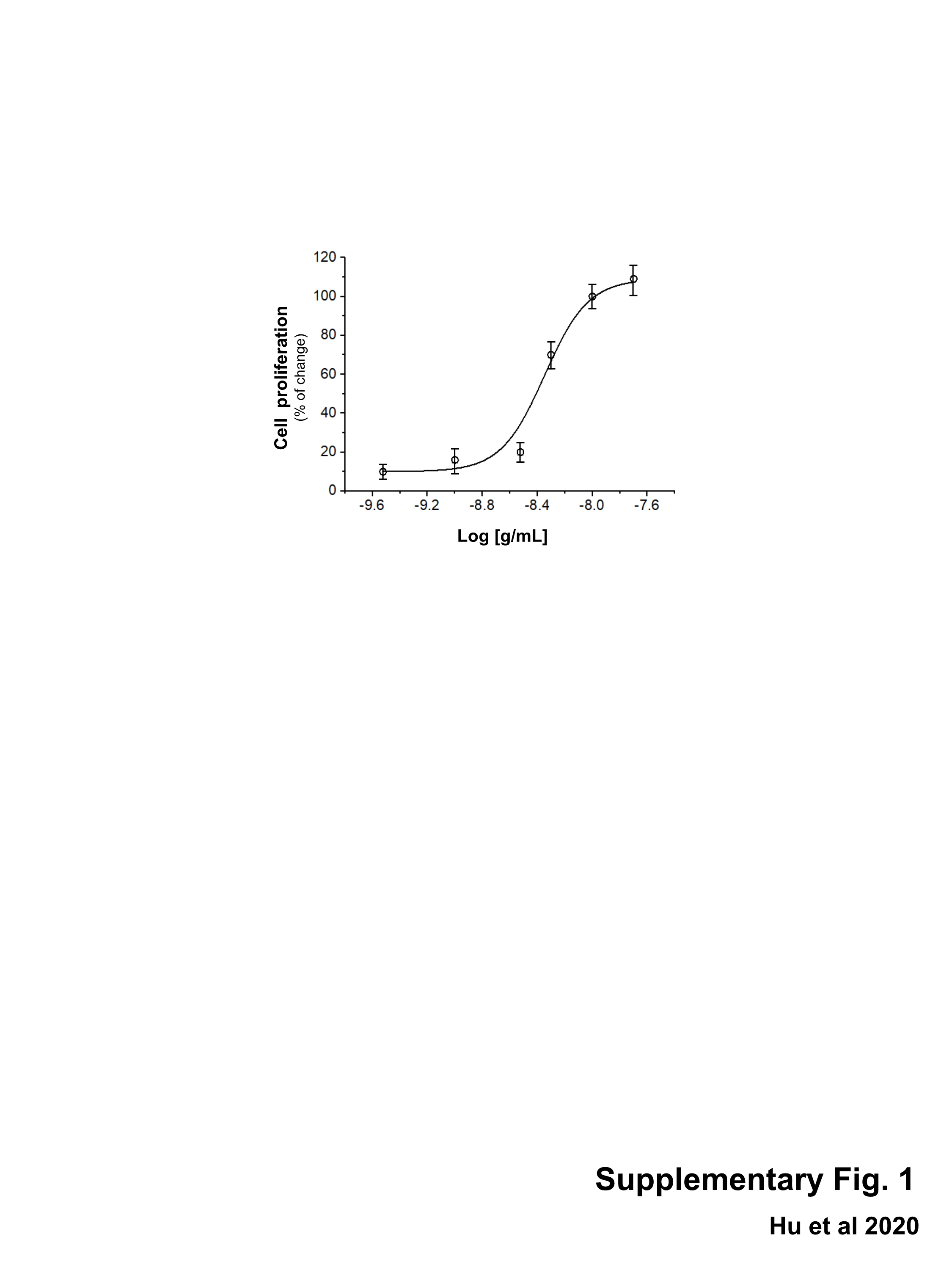

Supplement: Supplementary Figure 1 — VEGF promotes endothelial cell proliferation in a concentration-dependent manner. HUVECs were seeded onto a 96-well plate at 5,000 cells/well and treated with or without VEGF at different concentrations. The cell viability was assayed by MTT after 48 h of treatment. Data are demonstrated as Mean ± SEM of the percentage of change as compared to control group (no VEGF), where n = 5. [file Image_1.jpg]
